# Surgery of the Brain and Spinal Cord

**Published:** 1903-05-09

**Authors:** 


### Surgery of the Brain and Spinal Cord.

**A Cerebral Hydatid Cyst—Removal—Recovery.** Drs. Rennie and Crago[1] report this case. The patient was a man of 39, a resident of New South Wales, who had when first seen suffered from headache and vomiting for about three months, and was found on examination to have some left-sided paresis. He had had one or two attacks of momentary unconsciousness and occasional diplopia. A diagnosis of cerebral tumour was made and iodide of potash given, but all the symptoms got steadily worse and there was slight optic neuritis present. There was no anæsthesia and no alteration in the percussion note over the head, though the right side seemed rather the more tender. The localisation of the tumour was then made to the right cerebral hemisphere in the ascending frontal convolution in the site of the arm centre. As the patient was going down hill he was trephined over the area indicated, and about 4 ozs. of hydatid fluid withdrawn with a syringe. The disc of bone was then returned and the wound sutured. The symptoms were relieved by the proceeding and on the fourth day later the wound was again opened up under anæsthesia, the dura mater incised and a hydatid cyst pulled out with forceps complete. Its wall was found close under the surface of the brain. Owing to bleeding from the pia mater the wound had to be packed with gauze. The patient recovered well and was reported 18 months later as being in good health. The authors strongly recommend this method of operating *à deux temps*.

**The Hartley-Krause Flap in Hæmorrhage from the Middle Meningeal Artery.**—Plummer[2] relates two cases in which he used this flap. Hartley and Krause, working independently, devised an osteoplastic resection of the skull for resecting the trigeminal nerve intracranially. The flap in the one case was omega-shaped, and in the other more or less uterus-shaped, the base in each case being at the zygoma. The bone was cut round with a chisel and turned down with the soft parts. Plummer points out that we have in this flap the only method fulfilling all the requirements for an ideal exposure of the middle meningeal artery and its branches, and he then relates two cases in which he practised it. The symptoms in each case were typical of meningeal hæmorrhage and the flap exposed the hæmatoma, but in the second case more bone had to be removed posteriorly to remove all the blood clot. The first case died and post-mortem hæmorrhage was found in the fourth ventricle. The other case recovered, and the author confirms Krönlein's observation that the result of operation for hæmatoma resulting from meningeal hæmorrhage is generally favourable, unless complicated by simultaneous injuries resulting from the same cause as the hæmorrhage.

**Intracranial Neurectomy by a Simplified Method.** Dr. Abbe[3] described this. After pointing out what a serious operation it was by the Hartley-Krause method, and what a length of time it took, he said that he had recently adopted the method of making a vertical incision in the temporal region just within the hair line. He then chiselled a little hole through the thin portion of the temporal bone, and with the rongeurs gnawed away a section of bone about an inch and a half in diameter. Arterial hæmorrhage was eliminated by previous ligature of the external carotid; venous oozing was controlled by the pressure of gauze plugs covered with sterile rubber tissue. On exposing the brain, instead of removing the Gasserian ganglion the interior and superior maxillary branches of the fifth nerve were dissected out, and about half an inch of each removed on the inner side of the foramina rotunda and ovale. These two foramina of exit were then covered with a piece of rubber tissue which was allowed to remain permanently. Dr. Abbe said he had done this operation seven times. In all the neuralgia was of the most inveterate type, and in all the relief was prompt and, so far, permanent. The first case was operated on nearly seven years ago. The advantages of this operation over the Hartley-Krause were that it could be done much more rapidly, it was followed by less shock and hæmorrhage and that it was not followed by wasting of the tissues in the temporal region. In the discussion which followed, Dr. Weir thought that to attack the nerve on the side distal to the ganglion was a step in the wrong direction, and that unless the ganglion was destroyed the probability of a recurrence must always be feared. Dr. Dana thought the ganglion was always eventually involved, and that in old cases it was always necessary to remove it. He attributed Dr. Abbe's success to the fact that his operations were done comparatively early in the course of the disease, or at least before the ganglion had become much affected.

**Illustrative Cases of Gunshot Wounds of the Skull and Brain.**—Irvine[4] writes from an experience of 30 cases in South Africa. He starts by pointing out that these injuries account for a very large proportion—probably 60 per cent.—of those killed in action. The factors determining the nature and gravity of gunshot injuries of the skull and brain are mainly two: (1) the range at which the wound is inflicted, and (2) the region of the skull and brain involved; fractures of the base being of course more grave than those of the vertex. A classification is made into (1) gunshot injuries at close range; (2) gunshot injuries at medium ranges; and (3) cases in which the bullet is lodged in the cranial cavity. Two cases are quoted illustrative of the first variety. In the first of these the muzzle of the carbine was probably in contact with the head, and the explosive effect of the cordite was superadded to the effect of the bullet. The top of the head was practically blown off. In the second case the distance was but three paces and the injuries were much less than might have been expected. The bullet was from a Lee Metford. The extrance hole on the malar bone was small and clean without any fissuring, the exit admitting the index finger and showing circular fissuring around it. Death was instantaneous. If the apertures of entry and exit are close together a fissured fracture is likely to connect the two. If the bullet is distorted in its course there may be a large aperture of exit suggesting an "expanding" bullet but this is much more likely to occur in the limbs than in the skull. In the latter, at distances of 5 to 50 yards, there may be great local destruction and much fissuring but the general integrity of the scalp and cranium is preserved. Whatever the range, all gunshot fractures of the base involving the middle or posterior fossa with injury to the base of the brain are immediately or ultimately fatal. The medium ranges are given as from 500 to 800 yards, and the injuries occurring at these distances fall into two main classes, those causing depressed or gutter fractures and those causing complete perforation of the cranial cavity. Four cases of gutter fracture are referred to in only one of which were there any cerebral symptoms—they were all operated on and all recovered. Those causing complete perforation are subdivided into superficial and deep, the former travelling close under the skull are usually accompanied by fissuring, the cortex is ploughed through, the grey matter much damaged and cerebral irritability well marked. The superior longitudinal sinus is liable to be injured and hemiplegia to come on. In the deep variety the injury to bone and cortex is much lessened, the apertures on the bone are usually small and similar and there is an absence of fissuring. The brain is lacerated along the bullet track and the symptoms are those of cerebral irritation. Paralytic symptoms are less common than in the superficial variety. Nine cases are referred to of which eight were operated on, seven recovered, and two of the operation cases died probably of septic encephalitis. Dr. Irvine points out that the aperture of entry should be examined first and the aperture of exit after if the patient's condition admits. The last variety where the bullet is retained occurs from injuries at long ranges or ricochets. They are usually fatal. One fatal case is quoted where operation was performed but the bullet not found. The question of exploratory operation must depend on the individual circumstances of each case. If there are localising symptoms suggesting that the bullet may be accessible its removal should be attempted.

**The Results of the Operative Treatment of Spinal Tumours.**—Williamson [5] classifies spinal tumours into (1) those in the vertebræ (the cord symptoms being due to indirect pressure); (2) outside the dura mater —extra-dural meningeal tumour; (3) within the dura mater—intradural meningeal tumours; (4) within the cord substance, intramedullary. The tumours of the first and fourth group are unsuitable for surgical treatment, operation only being indicated in the meningeal growths. Tumours of the vertebræ, causing secondary pressure on the cord are probably more frequent than all the others put together. Tubercle forms the commonest tumour but is usually found associated with tubercular disease elsewhere and will rarely require consideration with reference to operative treatment. Almost every known variety of tumour has been found, but sarcomata and hydatid cysts were much the most common, fibromata and myxomata coming next. A table of 24 successful cases is given. When once a tumour of the cord is diagnosed there should be no delay in operation. Iodide of potash will probably be useless, as gummata of the cord are extremely rare. For operative treatment to be successful not only must a correct diagnosis of tumour be made, but the growth must be exactly localised by the upper limit of motor and sensory symptoms. So often has the tumour been found higher than was anticipated by the symptoms that Starr concludes that in operating a level of the cord should be exposed four inches higher than the sensory symptoms indicate, and if no tumour is found that the wound should be enlarged upwards.

[1] Australasian Med. Gaz., Nov. 20, 1902. [2] Annals of Surg., Oct. 1902. [3] Med. Rec., Jan. 17, 1903. [4] Lancet, Oct. 25, 1902. [5] Med. Chron., Sept. 1902.

*(To be continued.)*

